# Orally administered intelligent self-ablating nanoparticles: a new approach to improve drug cellular uptake and intestinal absorption

**DOI:** 10.1080/10717544.2021.2023704

**Published:** 2022-01-17

**Authors:** Yanzi Liang, Ruihuan Ding, Huihui Wang, Lanze Liu, Jibiao He, Yuping Tao, Zhenyu Zhao, Jie Zhang, Aiping Wang, Kaoxiang Sun, Youxin Li, Yanan Shi

**Affiliations:** a School of Pharmacy, Key Laboratory of Molecular Pharmacology and Drug Evaluation (Yantai University), Ministry of Education, Collaborative Innovation Center of Advanced Drug Delivery System and Biotech Drugs in Universities of Shandong, Yantai University, Yantai, P. R. China; b School of Life Science, Yantai University, Yantai, P. R. China; c State Key Laboratory of Long-acting and Targeting Drug Delivery System, Luye Pharmaceutical Co., Ltd., Yantai, P. R. China

**Keywords:** Oral drug delivery, zwitterionic materials, self-ablating nanoparticle, cellular uptake, intestinal absorption

## Abstract

Oral drug delivery to treat diabetes is being increasingly researched. The mucus and the epithelial cell layers hinder drug delivery. We designed a self-ablating nanoparticle to achieve smart oral delivery to overcome the gastrointestinal barrier. We used the zwitterionic dilauroyl phosphatidylcholine, which exhibits a high affinity toward Oligopeptide transporter 1, to modify poly(lactic-co-glycolic acid) nanoparticles and load hemagglutinin-2 peptide to facilitate its escape from lysosomes. Nanoparticles exhibit a core–shell structure, the lipid layer is degraded by the lysosomes when the nanoparticles are captured by lysosomes, then the inner core of the nanoparticles gets exposed. The results revealed that the self-ablating nanoparticles exhibited higher encapsulation ability than the self-assembled nanoparticles (77% vs 64%) and with better stability. Quantitative cellular uptake, cellular uptake mechanisms, and trans-monolayer cellular were studied, and the results revealed that the cellular uptake achieved using the self-ablating nanoparticles was higher than self-assembling nanoparticles, and the number of uptake pathways via which the self-ablating nanoparticles functioned were higher than the self-assembling nanoparticles. Intestinal mucus permeation, *in vivo* intestinal circulation, was studied, and the results revealed that the small self-assembling nanoparticles exhibit a good extent of intestinal uptake in the presence of mucus. *In vitro* flip-flop, intestinal circulation revealed that the uptake of the self-ablating nanoparticles was 1.20 times higher than the self-assembled nanoparticles. Pharmacokinetic study and the pharmacodynamic study showed that the bioavailability and hypoglycemic effect of self-ablating nanoparticles were better than self-assembled nanoparticles.

## Introduction

Oral drug administration is the most commonly used method of drug administration as the drugs used during the method are portable. The harsh acidic environment of the stomach, extensive enzymatic degradation in the presence of various enzymes, and the process of mucus clearance reduce the efficiency of the method (Fan et al., 2018). Therefore, nanocarriers designed for oral administration should be able to promote mucus permeation and epithelial absorption, although these two processes require significantly different carrier properties (Liu et al., 2016; Drucker, 2020). Hence, oral drug delivery systems are being increasingly studied. Commonly used Polyethylene glycol-modified nanoparticles have hydrophilic and near-neutrally charged surfaces that reduce the mucus adhesion by hindering hydrophobic or electrostatic interactions (Khutoryanskiy, 2018; Nie et al., 2019). It has been recently reported that a zwitterionic carrier platform can be used to simultaneously surmount the mucus and epithelial barriers. The use of zwitterions significantly increases the rate of protein payload transport. Moreover, the zwitterionic systems do not exhibit immune response, unlike Polyethylene glycol (PEG) (Xu et al., 2018; Debayle et al., 2019; Han et al., 2020). Zwitterionic polymers that are electrically neutral but bear identical cationic and anionic groups have attracted immense attention from researchers working in the field of biomedicine. Phosphorylcholine, carboxybetaine, and sulfobetaine are the most widely used systems (Peng et al., 2020; Bevilacqua et al., 2021).

The biggest obstacle faced by nanoparticles that have passed through the mucus layer is presented by the epithelial cell layer. Often, only the cytotoxicity of nanocarriers and their cargoes is studied, and the cellular uptake achieved by specific nanocarriers is not studied. The pathways of nanoparticles are internalized by cells are primarily clathrin-mediated endocytosis, macropinocytosis, and lipid raft-mediated endocytosis. The pathways are influenced by the physical and chemical properties (such as size, shape, and nature of surface modifications) of the nanoparticles (Kim et al., 2018; Sun et al., 2019; Lei et al., 2021). The interaction between nanoparticles and cell membranes is largely unknown and the fate of these nanoparticles and their cargo after internalization remains to be clarified. The nanoparticle-engulfing vesicles that enter the cell, further pass through various intracellular transport processes. First, cargo will be transported to early endosomes, which will mature into late endosomes. These late endosomes will fuze with pre-lysosomal vesicles to form lysosomes, which have an acidic and enzyme-rich environment for degradation (Hillaireau & Couvreur, 2009; Stewart et al., 2016). It is noteworthy that the drug is highly susceptible to degradation. Therefore, lysosomal escape strategies can be explored to optimize drug delivery. Hemagglutinin-2 peptide (HA_2_) is a viral membrane peptide that promotes the disruption of lysosomal membranes and facilitates the escape of particles from lysosomes (Selby et al., 2017; Xu et al., 2018; Smith et al., 2019).

We chose zwitterionic dilauroyl phosphatidylcholine (DLPC) as the shell of the self-ablating nanoparticles. The outer lipid shell encounters the complex enzymatic environment within the lysosomes and gets degraded following the capture of the nanoparticles by the lysosomes. Following this, the inner core gets exposed to the surroundings, and self-ablation is realized (Breiden & Sandhoff, 2021). We also prepared the Self-assembled nanoparticles at the same time. The cellular uptake and intestinal absorption effects of the two nanoparticles were compared. Drugs to treat type 2 diabetes are generally injected into the bodies of patients, causing inconveniences. Hence, it is important to develop drugs that can be orally administered. We used liraglutide, a glucagon-like peptide-1 (GLP-1) analog, as a model drug to study the oral delivery absorption effects generated of the nanoparticles (Alexopoulos & Buse, 2019; Muller et al., 2019; Choxi et al., 2020). First, self-ablating nanoparticles (LNPS) were prepared following a combination of the emulsifying solvent evaporation and thin-film dispersion methods. The nanoparticles could be readily trapped by the lysosomes, and we proceeded to load HA_2_ into LNPS during the hydration step of the thin-film dispersion method and obtain H-LNPS. DLPC that self-assembled on the surface of poly(lactic-co-glycolic acid) (PLGA) exploiting hydrophobic interactions to form Self-assembled nanoparticles (D-NPS) (Shan et al., 2016; Li et al., 2020). We determined the cellular uptake achieved by conducting cellular uptake imaging studies and trans-cellular monolayer assays. We also used the flow cytometry technique to study cellular uptake. The intestinal uptake of nanoparticles was examined by conducting rat mucus permeation, *in vivo* intestinal circulation, and *in vitro* flip-flop intestinal circulation studies. The bioavailability and hypoglycemic effect of the nanoparticles were investigated by pharmacokinetic study and pharmacodynamic study.

## Materials and methods

### Materials

PLGA (MW: 20 KDa; 50:50; termination group:hydroxyl group) was purchased from PolySciTech (West Lafayette, IN, USA). DLPC was purchased from Aladdin Industrial (Shanghai, China). 3-(2′-Benzothiazolyl)-7-diethylaminocoumarin (coumarin-6; C-6) was purchased from Aladdin Industrial (Shanghai, China). FITC-liraglutide was purchased from Shanghai Qiangyao Biochemical Co. Ltd. (Shanghai, China). HA_2_ was purchased from Shanghai Qiangyao Biochemical Co. Ltd. (Shanghai, China). Sprague–Dawley (SD) rats were purchased from GemPharmatech Co., Ltd. (Jiangsu, China).

### Preparation of nanoparticles

PLGA (2.0 mg), DLPC (1.0 mg), and liraglutide (0.5 mg) were dissolved in 0.2 mL of Dimethyl sulfoxide (DMSO). The mixture was slowly dropped into 5.0 mL of water under conditions of high-speed stirring (1000 rpm, 2 h) to obtain D-NPS. Finally, the sample was dialyzed to remove all DMSO (Shan et al., 2017).

Firstly, PLGA nanoparticles were fabricated following an emulsification–evaporation method. PLGA (2.0 mg) was dissolved in 1.0 mL of dichloromethane, and liraglutide (0.5 mg) was dissolved in 50 μL of water. The mixture was emulsified in an ice water bath for 30 s. The ultrasonic probe (SCIENTZ-IID, Ningbo Scientz Biotechnology Co., Ltd.) was set at 300 W. Subsequently, it was mixed with 10.0 mL of PVA solution (1%, w/v), and the mixture was sonicated for 30 s to obtain the W/O/W double emulsion. Dichloromethane was removed by stirring at room temperature (25 °C). DLPC and cholesterol were dissolved in 2.0 mL of anhydrous ethanol (mass ratio: 4:1). The solvent was evaporated using a rotary evaporator to form a lipid film. Subsequently, the films were hydrated with 10.0 mL of PLGA nanosolution and sonicated for 30 s to obtain the LNPs. The 0.3 mg HA_2_ peptide was introduced during hydration to prepare H-LNPS. The morphology of the NPs was observed using the transmission electron microscopy (TEM) technique using a JEM-1230 system (Jeol, Tokyo, Japan). The size and zeta potential were characterized using a Malvern Particle Size Analyzer.

The concentration of liraglutide was determined using the high-performance liquid chromatography technique (HPLC; Agilent Technologies), the Eclipse XDB-C18 column (4.6 mm × 250 mm; 5 µm) (Pedaprolu et al., 2016)was used. The eluent consisted of phase A and phase B. Phase A consisted of methanol and acetonitrile (80:20, v/v), and phase B consisted of 0.04% phosphoric acid solution. The flow rate was maintained at 1 mL/min, and the detection wavelength was 215 nm (elution conditions: A:B = 75:25).

The encapsulation efficiency of the liraglutide-loaded nanoparticles were calculated according to the following formula:

$$\text{Encapsulation efficiency }(\%)\;=\frac{\text{Total amount of liraglutide }-\text{ Free liraglutide}}{\text{Total amount of liraglutide}}\times 100$$

### Stability and *in vitro* release

#### Physical stability of the nanocapsules

The physical stability of the nanoparticles was investigated. The nanoparticles were dispersed in PBS at room temperature (25 °C), and the particle size and PDI values were determined in 1 week.

#### *In vitro* release

The drug release performances were studied in simulated gastric fluid (pH = 1.2) and simulated intestinal fluid (pH = 7.4). The nano-solution (1 mg/mL) was placed in a dialysis bag (100KD, Spectrum) at 37 °C. It was placed in the simulated gastric fluid (20 mL) for the first 2 h and then transferred to the simulated intestinal fluid (20 mL, total treatment time: 24 h). At specific time intervals, 1.0 mL of the solution was removed from the release medium, and an equal volume of the fresh release medium was added to the system. The samples were analyzed using the HPLC technique, and the cumulative release profile was determined.

### Cell studies

#### Cytotoxicity: methods

The cell cytotoxicity was detected by conducting MTT(Thiazolyl Blue Tetrazolium Bromide) assays. Caco-2 cells in a logarithmic growing period were seeded at a density of 3000 cells per well in 96-well plates (incubation conditions: 37 °C, 5% CO_2_, 24 h). Subsequently, the medium was replaced with the different concentrations of the NPs solutions (50–1000 μg/mL). The control group was cells incubated with a culture medium. The culture medium was used as a background group. Following 24 or 48 h of incubation, the medium was removed, and MTT (20 μL) was added into the system. The sample was incubated for 4 h. Following this, DMSO (200 μL) was introduced to replace MTT, and the absorbance assay was performed.

$$\text{Cell viability }(\%)\;=\;\frac{A-A0}{A1-A0}\times 100$$

*A* is the absorbance of the nano group, *A*1 is the absorbance of the control group, *A*0 is the absorbance of the background group.

#### Cellular uptake studies

The extent of cellular uptake achieved was evaluated using the flow cytometry technique and a high-resolution live-cell imaging system. Caco-2 cells were seeded (density: 3 × 10^4^ cells/well) in 24-well plates, and the cells were incubated for 48 h. C6 was used to label the nanoparticles. C6 is loaded in the same way as the drug. C6-labeled nanoparticles (100 ng/mL) were added to each well. The cells were incubated for 1, 2, and 4 h, following which the cells were washed with PBS and then fixed using 4% formaldehyde. The nuclei were stained using DAPI and observed using a high-resolution live-cell imaging system. Caco-2 cells were seeded in 6-well plates (density: 3 × 10^5^ cells per well) and incubated for 48 h. C6-labeled nanoparticles (4 ng/mL) were added to each well and incubated for 1, 2, and 4 h. The free C6 was used as a control group. Following trypsinization, the cells were collected following the process of centrifugation (1000 rpm, 5 min) and washed with PBS. Finally, fluorescence intensity was recorded and quantified using the flow cytometry technique.

#### Investigation of the cellular uptake mechanism

Various cell pathway inhibitors were used to understand the method of internalization of the nanoparticles. Caco-2 cells in their logarithmic growing period were seeded at 3 × 10^5^ cells/well in 24-well plates and incubated for 48 h. The inhibitors chlorpromazine (30 µmol/L), 5-(N-Ethyl-N-isopropyl)amiloride (EIPA; 20 µmol/L), and Methyl-β-cyclodextrin (M-β-CD; 2.5 mM) were then added to the systems, which were then incubated for 0.5 h. For the control group was treated with a culture medium instead of an inhibitor. Then, fluorescent nanoparticles were added to the cells, which were then incubated for 1 h. Following the treatment method described above, the fluorescence intensities of the cells were measured using the flow cytometry technique.

We chose the oligopeptide transporter 1 (PEPT1) inhibitor glycylsarcosine (Gly-Sar) to study the effect of PEPT1 on cellular uptake. As described above, Caco-2 cells were seeded at 3 × 10^5^ cells/well in 24-well plates and incubated for 48 h. The inhibitors Gly-Sar (20 µmol/L) were then added to the systems, which were then incubated for 0.5 h. For the control group was treated with a Culture medium instead of an inhibitor. Then, fluorescent nanoparticles were added to the cells, which were then incubated for 1 h. Following the treatment method described above, the fluorescence intensities of the cells were measured using the flow cytometry technique (Wu et al., 2017; Zheng et al., 2018).

#### Lysosomal escape

Caco-2 cells were seeded in a 24-well plate (density: 3 × 10^4^ cells/well) and cultured for 48 h. The fluorescently labeled nanoparticles were incubated with the Caco-2 cells for 2 h. Following this, the cells were washed using PBS to remove the non-internalized nanoparticles. The cells were stained using Lysozyme Tracking Red (200 µL) and fixed with paraformaldehyde. Subsequently, DAPI was added for nuclear staining. The cells were rewashed and imaged using a multi-function cell microwell imager.

#### Transmembrane transport

We constructed an *in vitro* model of simulated gastrointestinal mucus and epithelial layer using co-cultured Caco-2 and HT29-MTX cells (Zhang et al., 2015). A mixture of Caco-2 and HT29-MTX cells (density: 2 × 10^5^ cells/well, mixing ratio: 8:2) were inoculated into the transwell and cultured at 37 °C in an incubator containing 5% CO_2_ until the transepithelial resistance reached 300–500 Ω cm^2^. The culture medium was replaced with preheated Hank’s buffered salt solution (HBSS) and equilibrated for 30 min. Following this, HBSS was replaced with FITC-labeled nanoparticles in the upper chamber. Free FITC-liraglutide was used as a control group. Some amount of the solution (0.5 mL) was taken out from the lower chamber at 0.5, 1, 2, and 3 h, respectively, and the system was supplemented with the same volume of preheated HBSS solution. The cumulative transport of FITC–liraglutide was determined (Zhang et al., 2018).

### Intestinal absorption studies

#### Mucus penetration and aggregation

Fresh mucus from the rat intestine was evenly distributed in the transwell and balanced at 37 °C for 30 min. Following this, PBS (1000 µL) was added to the lower chamber. Subsequently, the FITC-labeled nanosolution (500 µL) was added to the upper chamber (60 µg/mL). Free FITC-liraglutide was used as a control group. At the time points of 15 min, 0.5 h, 1 h, 1.5 h, and 2 h, 200 µL of the solution was removed from the lower cavity, and supplemented with 200 µL of fresh PBS. The apparent permeability coefficient (*P*_app_) was calculated, and the mucus retention abilities of the nanoparticles were determined.

*P*_app_ was calculated as follows:

$$P_{\mathrm{app}}=\mathrm{dQ}/\mathrm{dt}\times 1/(A\times C0)$$

Where dQ is the cumulative amount of NPs that permeate the basolateral side, A is the area of the monolayer (cm^2^), and C0 is the initial concentration of NPs on the top side. dQ/dt is the slope of the linear relationship between the cumulative amount of NPs transported (μg) and time (min).

#### *In vivo* intestinal circulation

SD rats aged 6 weeks were allowed to fast overnight. Then, the rats were anesthetized by intraperitoneally injecting chloral hydrate. An abdominal incision was made to expose the small intestine. The small intestine was ligated every 4 cm to form a ring. Fluorescent-labeled nanoparticles were injected into the intestinal ring to simulate the absorption of drugs in the intestinal environment. Free FITC-liraglutide was used as a control group. After 2 h, all intestinal rings were thoroughly washed to remove residual nanoparticles. The intestine was treated with a high-speed shear. Following the homogenization of the fragment, the fluorescence signal was recorded (Cheng et al., 2020).

#### *In vitro* flip-flop intestinal circulation

SD rats were fasted overnight and anesthetized using chloral hydrate. The small intestine of the rats was removed. The small intestine was divided into lengths of 5 cm and placed in Tyrode’s solution. Following the excision of the mesentery and lymph nodes, the small intestine was turned over and washed. Tyrode’s solution (0.5 mL) was injected into the ring, following which the two ends of the intestinal segment were bound tightly. The everted intestinal rings were placed in fluorescent-labeled nanoparticles and cultured at 37 °C for 3 h in an incubator containing 5% CO_2_. Free FITC-liraglutide was used as a control group. The intensity of the fluorescence signal of the liquid in the intestinal ring was recorded following incubation.

#### Pharmacokinetic study

SD rats (GemPharmatech Co., Ltd.) weighing 200–220 g were fasted overnight and randomly divided into five groups(*n* = 5/group): Liraglutide solution group, D-NPS group, LNPS group, H-LNPS group, Liraglutide subcutaneous injection group. The first four groups were administered orally. After a single administration, blood was collected and centrifuged, and serum was collected. An ELISA Kit for liraglutide was used to measure the blood concentration of liraglutide. The assay was performed according to the instructions of the ELISA Kit for liraglutide. The processed samples were tested using a microplate reader (CYT5MV microplate reader, BioTek USA Botten Instruments Co., Ltd.), and the drug concentration was calculated by plotting the curve.

The relative bioavailability (BR) of the NPs was calculated according to the following formula:

$$B_{R}\;(\%)=\text{AUC}\;(i.g.)\times \text{dose}\;(s.c.)/\text{AUC}\;(s.c.)\times \text{Dose}\;(i.g.)\times 100\%$$

where AUC is the area under the curve, i.g. is intragastric administration, and s.c. is subcutaneous.

#### Pharmacodynamic study

##### Single dose study

Pharmacodynamic studies were performed using db/db mice (GemPharmatech Co., Ltd.). Mice were randomly divided into 6 groups (*n* = 5/group) and given saline, liraglutide solution, D-NPS, LNPS, H-LNPS (540 µg/kg), and liraglutide subcutaneously (54 µg/kg). After a single dose, blood was taken from the tail vein to measure blood glucose levels.

##### Multiple dose study

Mice were grouped as described above. The mice were administered once a day, and the blood glucose levels were measured 24 h after the administration of the drug. The mice were administered for 5 consecutive days, and the changes in blood glucose levels were checked.

### Statistical analysis

Data were expressed as mean ± standard deviation (SD). The statistical significance of the results was analyzed by conducting a two-tailed Student’s *t*-test. A value of *p* < .05 was considered statistically significant, and a value of *p* < .01 was considered highly significant.

## Results

### Characterization of the nanoparticles

**Figure 1.**
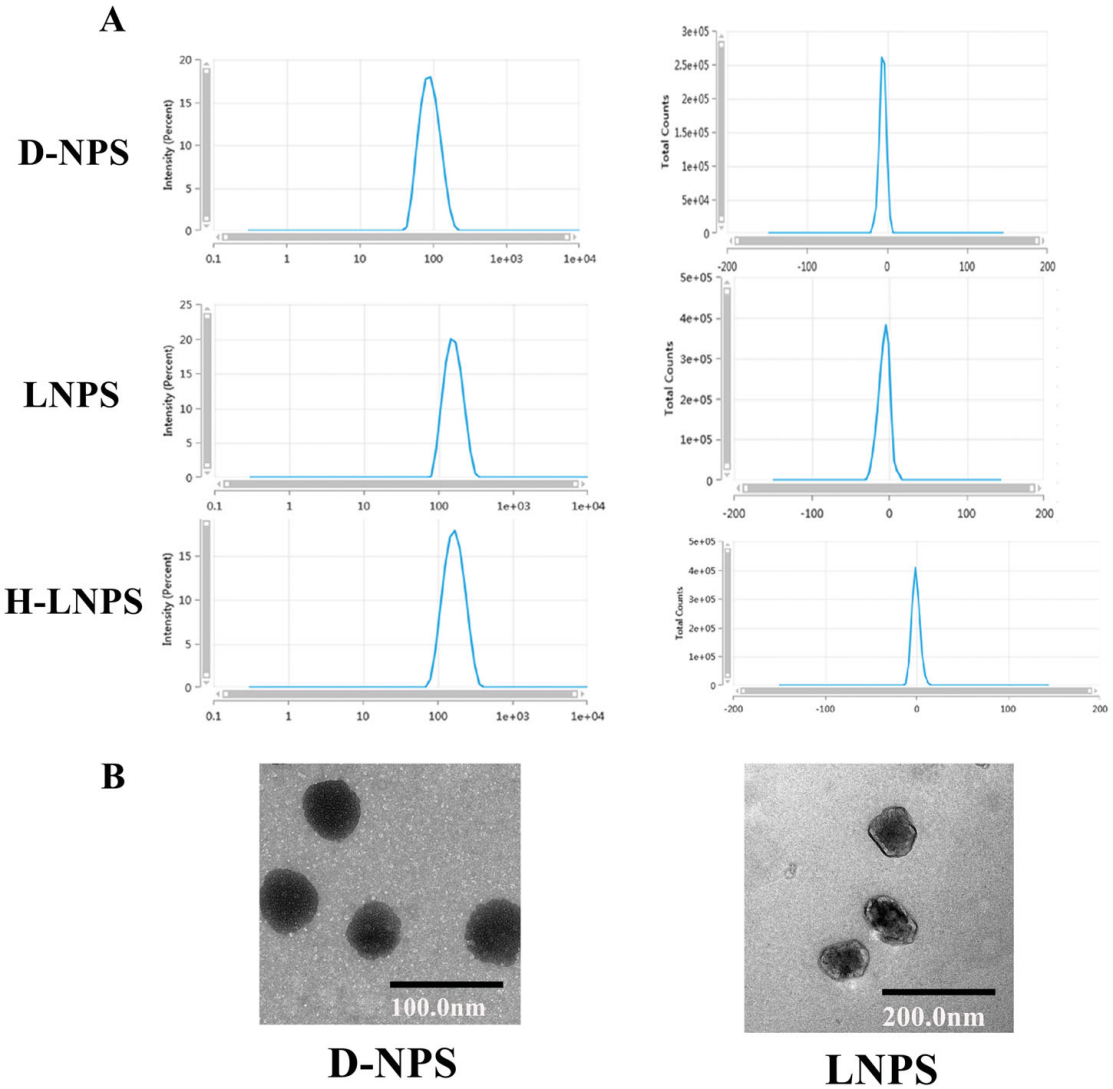
(A) Size and zeta potential map for nanoparticles. (B) Electron micrograph map of D-NPS and LNPS.

As shown in Table 1, the particle sizes of LNPS (148.17 ± 1.63 nm) and H-LNPS (152.21 ± 0.15 nm) were approximately twice the particle sizes of D-NPS (81.29 ± 3.26 nm). The zeta potentials were close to zero. D-NPS appeared spherical, and LNPS exhibited an irregular core–shell structure (Figure 1(B)).

**Table 1.** Size (nm), zeta potential (mV), encapsulation efficiency (%) of nanoparticles.

Formations	Size (nm)	Zeta (mV)	Encapsulation efficiency (%)
D-NPS	81.29 ± 3.26	−5.41 ± 0.85	63.99 ± 0.28
LNPS	148.17 ± 1.63	−2.21 ± 0.41	77.42 ± 1.55
H-LNPS	152.21 ± 0.15	−1.81 ± 0.62	75.31 ± 2.23

The encapsulation efficiency was determined using the HPLC. The encapsulation efficiency of D-NPS was approximately 64%, and the encapsulation efficiency of LNPS and H-LNPS was higher (>75%). The encapsulation rate of HA_2_ in H-LNPS is about 55%.

### Stability and *in vitro* release

**Figure 2.**
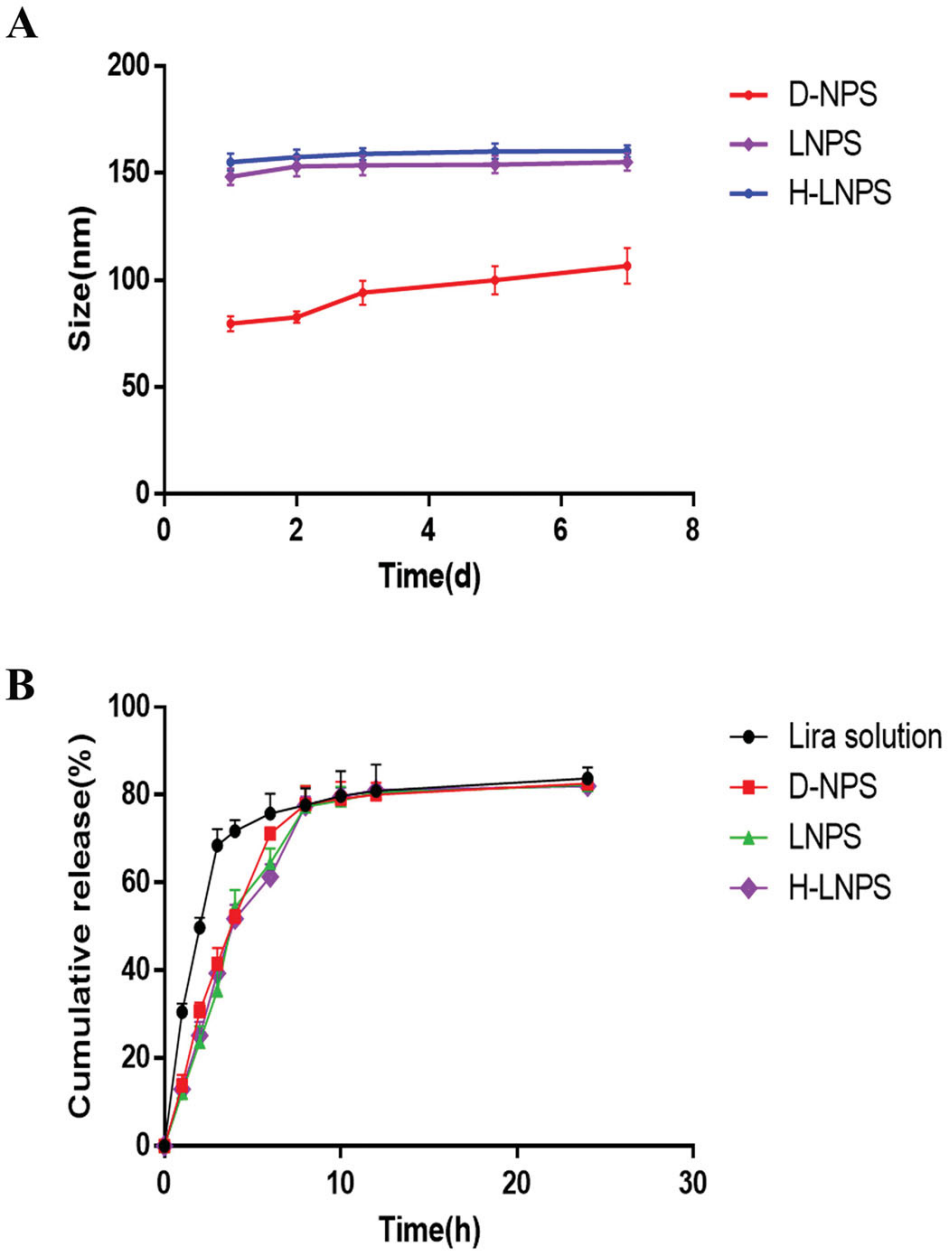
(A) Physical stability of the nanoparticles. (B) *In vitro* release of nanoparticles and liraglutide solution (first 2 h in simulated gastric fluid; 2–24 h in simulated intestinal fluid).

We observed the changes in the particle size and PDI of the nanoparticles over a period of 1 week. The particle size of D-NPS increased by 30 nm (Figure 2(A)), and the PDI increased from 0.12 to 0.26 within 1 week. It was also observed that the particle size and PDI for LNPS and H-LNPS remained basically unchanged. This proved that LNPS was more stable than D-NPS.

*In vitro* release experiments were performed under simulated gastrointestinal conditions. According to the results in Figure 2(B), it can be seen that the release of the free drug reached about 50% at 2 h, while the release of liraglutide coated by the carrier was only 20–30% at 2 h, indicating that the carrier coating has a slow-release effect on the drug release. The percentage release of the nanoparticles reached 80%. As the stability of the released liraglutide was influenced by the environment in the gastrointestinal fluid, 100% release could not be realized.

### Cytotoxicity

The extent of cell survival was recorded following the co-incubation with the nanoformulations (incubation times: 24 h and 48 h). The extent of cell survival was >80%, and the cell survival rate decreased as the concentration of the nanosolution increased. This indicated that D-NPS, LNPS, and H-LNPS were not highly toxic.

### Cellular uptake

Imaging studies were performed to determine the extent of cellular uptake achieved. Blue fluorescence indicated the nucleus, and green fluorescence indicated the nanoparticles. Time-dependent cellular uptake was observed for each group of nanoparticles (Figure 4(C)). The intensities of fluorescence (green fluorescence) recorded for LNPS and H-LNPS were higher than the D-NPS group.

The mode of nanoparticle entry was investigated by adding inhibitors. A significant reduction in cellular uptake in the presence of inhibitors (Figure 4(B)) was observed of LNPS and H-LNPS. This revealed that the nanoparticles entered the system via clathrin-mediated endocytosis, macropinocytosis, and lipid raft-mediated endocytosis pathways. The results also revealed that the PEPT1 also participated in the endocytic mechanism. The inhibitory effect of EIPA on D-NPS was not significant, indicating that the uptake pathway followed by D-NPS does not involve macropinocytosis.

The uptake of the cells was studied quantitatively using the flow cytometry technique. It can be concluded from Figure 4(A) that the intensity of the fluorescence recorded for H-LNPS and LNPS was stronger than the D-NPS group. This is consistent with the results presented in Figure 4(C).

### Monolayer cellular transport

We evaluated the ability of nanoparticles to pass through the epithelial cells using the Caco-2 and HT29-MTX cell lines as model systems. As seen in Figure 4(D), the Cumulative transport recorded for H-LNPS and LNPS was higher than the D-NPS group.

### Lysosomal escape

The lysosomal escape effect of nanoparticles loaded with HA2 was investigated. LNPS could be readily trapped by lysosomes (Figure 5). H-LNPS exhibited less co-localization with lysosomes, indicating that the HA2 peptide facilitated the escape of the nanoparticles from the lysosomes.

### Infiltration and aggregation of mucus

We spread rat intestinal mucus in the transwell to explore the mucus permeability of the nanoparticles. The mucus permeation coefficient for D-NPS was 1.24 times higher than that of LNPS and 1.32 times higher than that of H-LNPS (Table 2). The mucus permeation effect of D-NPS was better than that of the others. The retention of D-NPS was also found to be low (Figure 6(A)). This can be attributed to the smaller particle size of D-NPS. The small nanoparticles can readily penetrate the mucus, while the large nanoparticles are readily captured by the mucus. Thus, a size-dependent mucus penetration property was observed.

**Table 2.** *P*_app_ values of free FITC-liraglutide, D-NPS, LNPS, and H-LNPS when incubated with mucus.

Formations	*P*_app_ (cm/s) × 10^−7^
Free FITC-liraglutide	1.27 ± 0.11
D-NPS	4.24 ± 0.18
LNPS	3.41 ± 0.47
H-LNPS	3.21 ± 0.25

### Intestinal absorption

To better study the absorption of nanoparticles in the intestinal tract, we conducted *in vivo* intestinal circulation and *in vitro* flip-flop intestinal circulation experiments. As shown in Figure 6(B), the extent of absorption of D-NPS was slightly greater than LNPS and H-LNPS. The difference in the extent of absorption of LNPS and H-LNPS was small. Analysis of Figure 6(C) reveals that the extent of absorption of H-LNPS and LNPS was greater than the extent of absorption of D-NPS. The results revealed that during *in vivo* intestinal circulation in the presence of mucus, the extent of absorption of D-NPS was higher than H-LNPS and LNPS, and during *in vitro* flip-flop intestinal circulation, the extents of absorption of H-LNPS and LNPS were higher than D-NPS (attributable to the removal of the mucus layer) (Guo et al., 2021).

### Pharmacokinetics

Bioavailability is an important parameter that determines the pharmacokinetic effect of a drug. We investigated the bioavailability of liraglutide solution, D-NPS, LNPS, H-LNPS, and subcutaneous liraglutide. The blood concentration-time distribution curves of liraglutide are shown in Figure 7(A), and the related pharmacokinetic parameters are shown in Table 3.

**Table 3.** Pharmacokinetic parameters of liraglutide following administration of different liraglutide formulations.

	Lira (s.c.)	Lira (oral)	D-NPS	LNPS	H-LNPS
*C*_max_ (pg/mL)	185.27 ± 9.71	98.01 ± 8.22	122.50 ± 4.70	137.58 ± 11.87	156.79 ± 12.32
*T*_max_ (h)	12	2	10	14	14
AUC (pg h/mL)	1975.46 ± 108.95	1061.18 ± 56.55	1457.74 ± 129.54	1957.30 ± 254.69	2038.19 ± 114.09
BR (%)	100	5.37	7.38	9.91	10.32

The plasma concentration of liraglutide solution in the oral group was very low. The maximum blood concentration was reached at 12 h in the subcutaneous group and at 10 h in the D-NPS group, while the peak blood concentration was reached at 14 h in the oral LNPS and H-LNPS groups. Using subcutaneous administration as a control, the bioavailability of the oral H-LNPS group was 10.32%, that of LNPS was 9.91%, and that of D-NPS was 7.38%. The bioavailability of the oral liraglutide solution group was 5.37%. These data suggest that nanoparticle encapsulation improved the bioavailability of liraglutide via the oral route of administration.

### Pharmacodynamics

The hypoglycemic effect of different preparations was evaluated using db/db diabetic model mice. The hypoglycemic effects of saline, liraglutide solution, D-NPS, LNPS, and H-LNPS, and subcutaneous injection of liraglutide were observed in type 2 diabetic mice. As the results shown in Figure 7(B), free liraglutide solution and saline could not induce hypoglycemic effects because the free peptides were easily digested by proteases in the gastrointestinal tract. Subcutaneous injection of liraglutide solution significantly lowered the blood glucose value, reaching 77% of the initial value at 1 h and 63% of the initial value at 12 h. D-NPS reached 70% of the initial blood glucose value at 12 h, LNPS reached 64% of the initial value at 14 h, while H-LNPS had a more pronounced hypoglycemic effect (62%) at the same time point. Based on the above experimental results, it can be concluded that the use of encapsulation is an effective method to enhance the hypoglycemic effect.

The blood glucose levels after multiple administrations are shown in Figure 7(C). liraglutide solution and the saline group had no hypoglycemic effect after 5 repeated administrations. the hypoglycemic effect of H-LNPS group was better than that of the D-NPS group and LNPS group, and the blood glucose level of the H-LNPS group was able to maintain between 60% and 70%. The subcutaneous group also maintained blood glucose levels between 60% and 70%, but the overall effect was slightly better than that of H-LNPS.

## Discussion

The mucus and epithelial layers present in the gastrointestinal tract hinder the absorption of nanoparticles. The particles that can surmount the mucus barrier have properties that are different (and opposite) from the properties of the particles that can surmount the epithelial barrier. It has been reported that zwitterionic materials are better than PEG as hot mucus inert materials as they do not produce an immune response (Gao et al., 2021). Therefore, we chose the zwitterionic DLPC to fabricate a core–shell structured nanoparticle capable of self-ablation. The outer layer of phosphatidylcholine can be degraded by enzymes present in the lysosomes. This helps achieve self-ablation. During the process, the inner core gets exposed to the environment promoting intracellular delivery. We chose PLGA to prepare the core nanoparticles as this polymer is widely used for nano-delivery. PLGA nanoparticles were first prepared following the emulsification–volatilization method. Following this, DLPC was spin-distilled with cholesterol to form a film and hydrated with a nanosolution of PLGA to form LNPS following the thin-film dispersion method. We also surface-coated PLGA with DLPC exploiting self-assembling properties to obtain D-NPS. During the process of cellular uptake, nanoparticles were easily trapped by lysosomes. We also loaded HA_2_ onto LNPS to prepare H-LNPS that promoted lysosomal escape.

The core–shell structured LNPS nanoparticles were successfully fabricated, and the particle size was approximately 150 nm (Figure 1(B)). The particles were almost electrically neutral. Thus, they can efficiently penetrate the mucus layer. The rate of encapsulation recorded for LNPS was higher than D-NPS. *In vitro* release experiment showed that the nano group achieved a smoother and slower release pattern than the liraglutide solution group. This suggests that the drug encapsulated by the carrier achieves a slow release. The stability of the nanoparticles was examined by evaluating the particle size and PDI of the nanoparticles. It was observed that the particle size of D-NPS was variable, and the PDI increased, while the particle size and PDI recorded for LNPS and H-LNPS did not change significantly (Figure 2(A)). D-NPS was less stable than LNPS because the particle size was significantly small. The less stability could also be attributed to the high surface free energy that promoted aggregation. Lipid–polymer hybrid nanoparticles are characterized by the properties of polymer nanoparticles and liposomes. They exhibit high structural integrity, structural stability, and biocompatibility as they are characterized by a core–shell structure (Du et al., 2021; Sivadasan et al., 2021).

**Figure 3.**
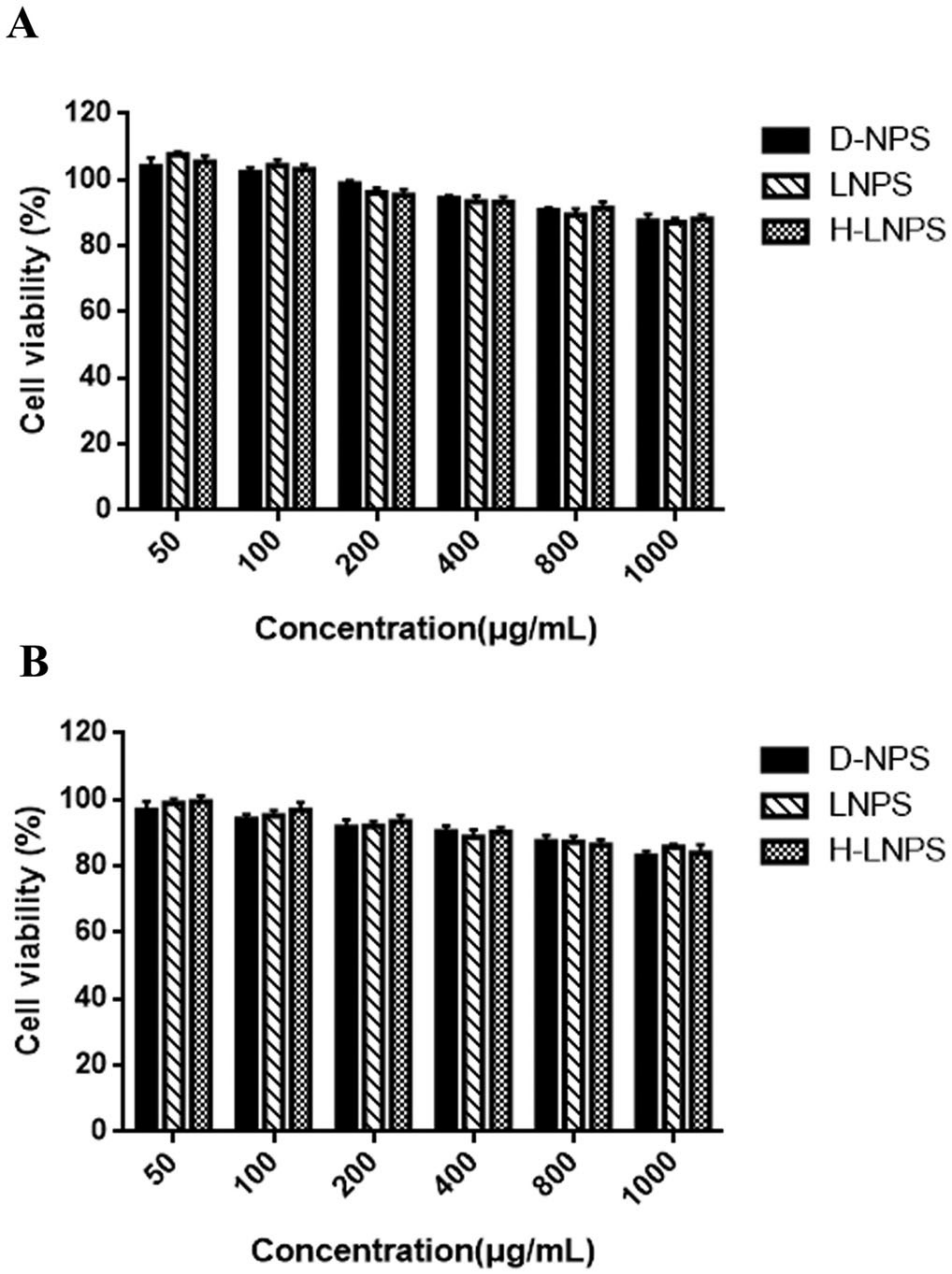
Cytotoxicity studies at 24 h (A) and 48 h (B).

**Figure 4.**
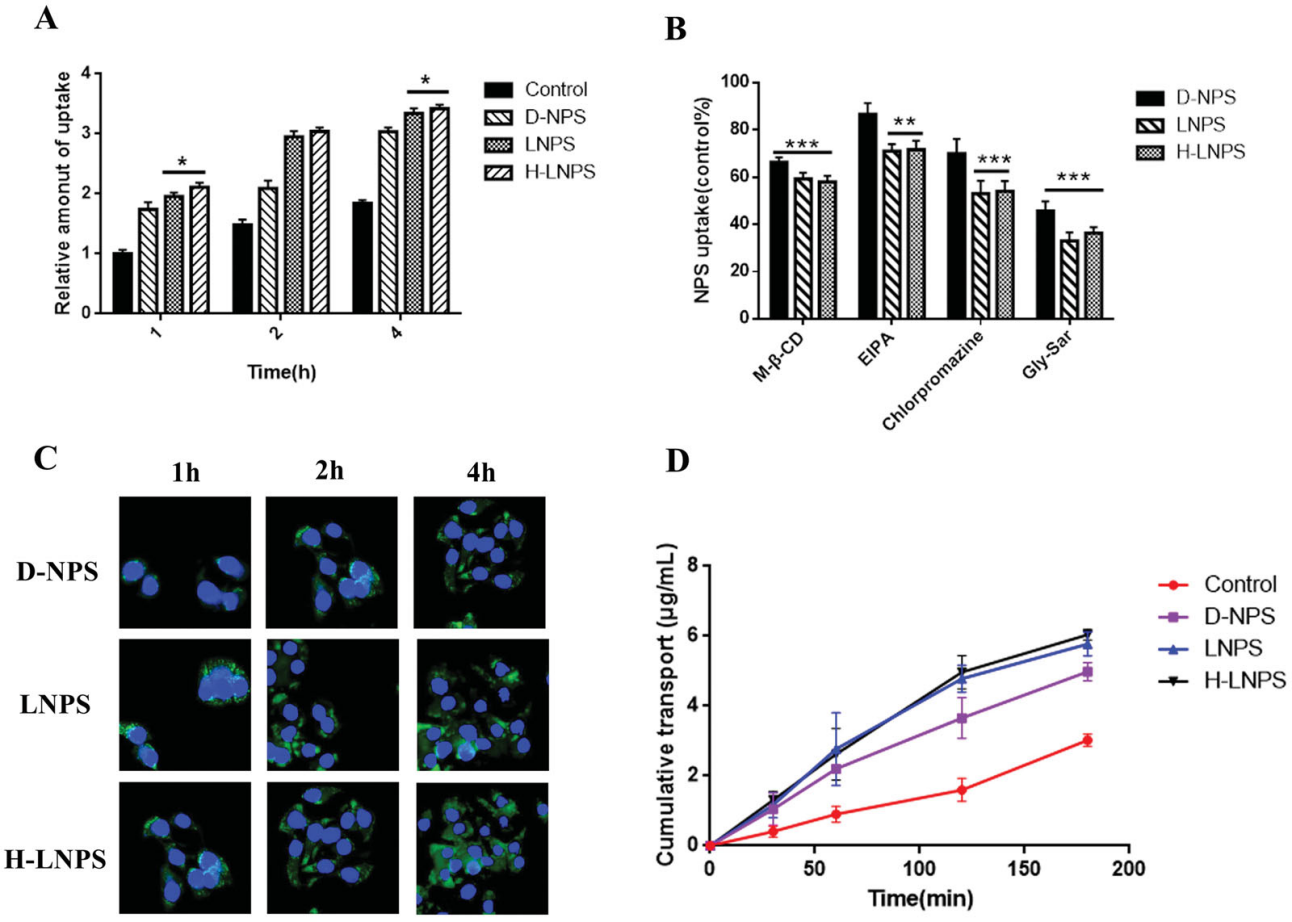
(A) Relative fluorescence intensity of coumarin-6 in cells. (B)Relative amounts of cell uptake of NPs in the presence of different endocytosis inhibitors. Using the control group as the benchmark of 100%. *p < .05; **p < .01; ***p < .001. (C) Cellular uptake map recorded for D-NPS, LNPS, and H-LNPS (blue fluorescence: nucleus; green fluorescence: nanoparticles). (D) Cumulative transport observed in the Caco-2 and HT29-MTX co-culture models.

The cytotoxicity of each group of nanoparticles was studied (Figure 3). It was observed that the nanoparticles were nontoxic. The extent of uptake achieved for LNPS and H-LNPS was better than D-NPS (Figure 4). The uptake pathways followed by self-assembled nanoparticles and self-ablating nanoparticles were investigated using specific uptake pathway inhibitors. Chlorpromazine, EIPA, and M-β-CD were used to examine clathrin-mediated endocytosis, macropinocytosis, and lipid raft-mediated endocytosis pathways, respectively. We also observed that DLPC exhibited a high affinity toward the PEPT1 transporter protein. Hence, Gly-Sar, an inhibitor of the PEPT1 transporter protein, was chosen to determine whether the entry of the nanoparticles into the cells involved the PEPT1 transporter protein. The uptake of LNPS and H-LNPS proceeded through clathrin-mediated endocytosis, macropinocytosis, and lipid raft-mediated endocytosis pathways. The PEPT1 transporter proteins also participated in the process (Yamada et al., 2015; Shan et al., 2016; Ran et al., 2018). It was also observed that the EIPA inhibition of D-NPS was weak, and therefore D-NPS did not enter the cells via micropinocytosis (Figure 4(B)). The uptake pathway is influenced by various properties of the nanoparticles. Particle size is one of the influential properties. Large nanoparticles are primarily transported via macropinocytosis. As D-NPS is small, macropinocytosis is an insignificant pathway for the entry of D-NPS. The epithelial cell permeability of nanoparticles was determined by constructing cell models and conducting trans-cellular monolayer experiments. The cumulative transport of H-LNPS and LNPS was higher than that of the D-NPS.

**Figure 5.**
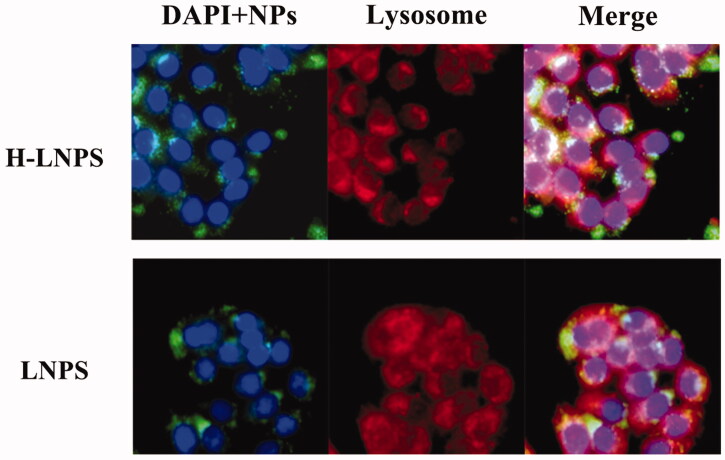
Co-localization images recorded for lysosomes of LNPS and H-LNPS (red: lysosomes; blue: nuclei; green: nanoparticles).

It was also observed that the nanoparticles could be readily captured by lysosomes and the loading of HA_2_ facilitated the escape of nanoparticles from lysosomes (Figure 5). Following the capture of the nanoparticles, the outermost lipid membrane gets degraded in the presence of the enzymes present inside the lysosome. Following this, the PLGA-containing nanocore and HA_2_ get exposed to the surrounding. Subsequently, HA_2_ acts on the lysosome to disrupt the lysosomal membrane and facilitates the escape of the PLGA nanoparticles from the lysosomes.

**Figure 6.**
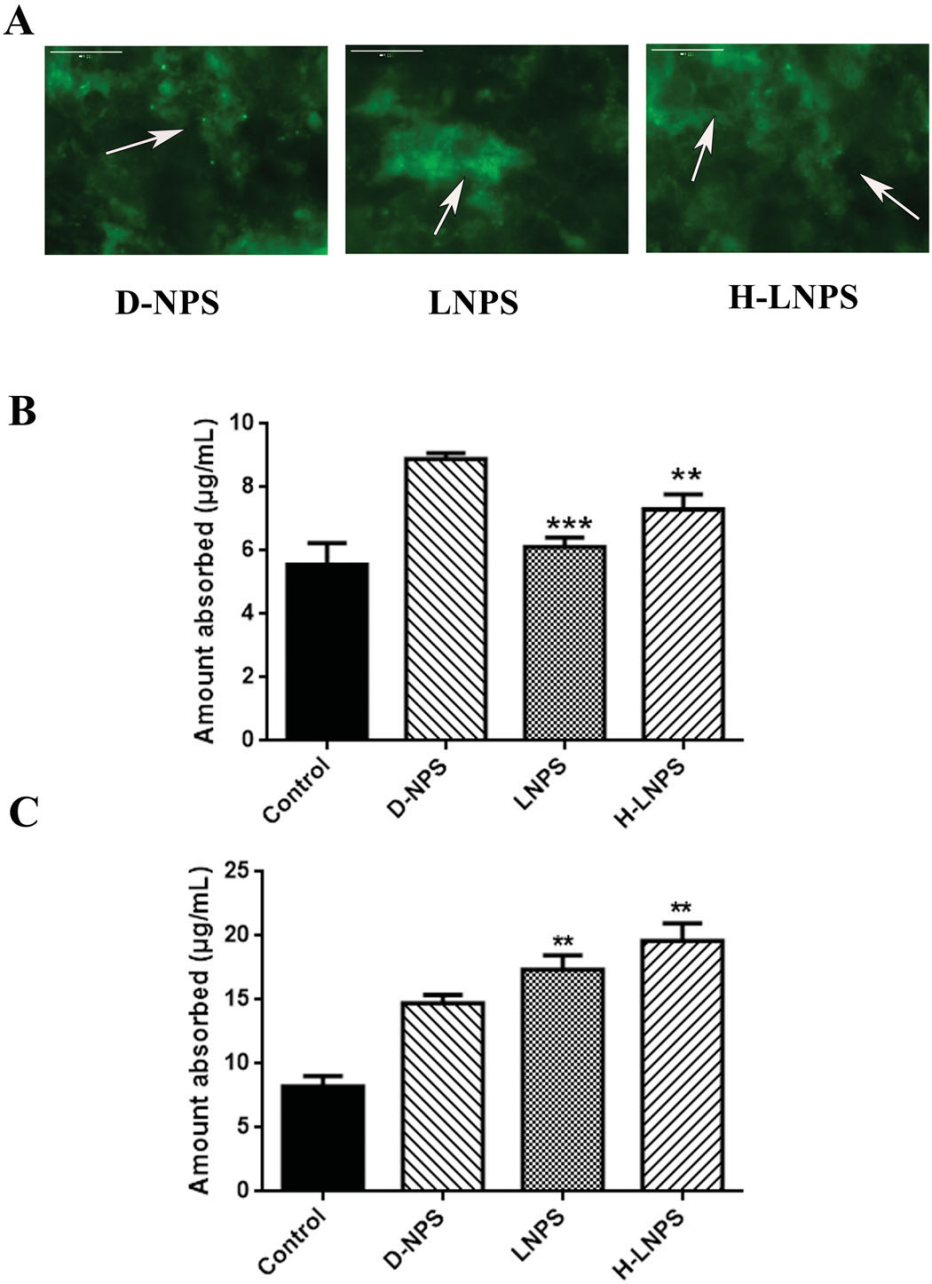
(A) Retention of nanoparticles in mucus. (B) Absorption of nanoparticles during the *in vivo* intestinal circulation. (C) Absorption of nanoparticles during the *in vitro* flip-flop intestinal circulation. Compared with the D-NPS group, **p < .01; ***p < .001.

Mucus permeation experiments were performed by scraping intestinal mucus from rats. A high permeation coefficient and low retention ability were observed for D-NPS (Table 2 and Figure 6(A)). *In vivo* intestinal circulation experiments were conducted, and it was observed that the extent of uptake recorded for D-NPS was higher than H-LNPS and LNPS. This could be attributed to the small particle size of D-NPS. The small particles can readily penetrate the mucus layer under hydrophilic and electrically neutral nano conditions. Better penetration was observed for H-LNPS and LNPS (compared to D-NPS) when the *in vitro* flip-flop intestinal circulation experiments were conducted. This could be attributed to the fact that the flipped loop relieved the influence of the mucus layer. This also reflects the high extent of cellular uptake achieved for H-LNPS and LNPS.

**Figure 7.**
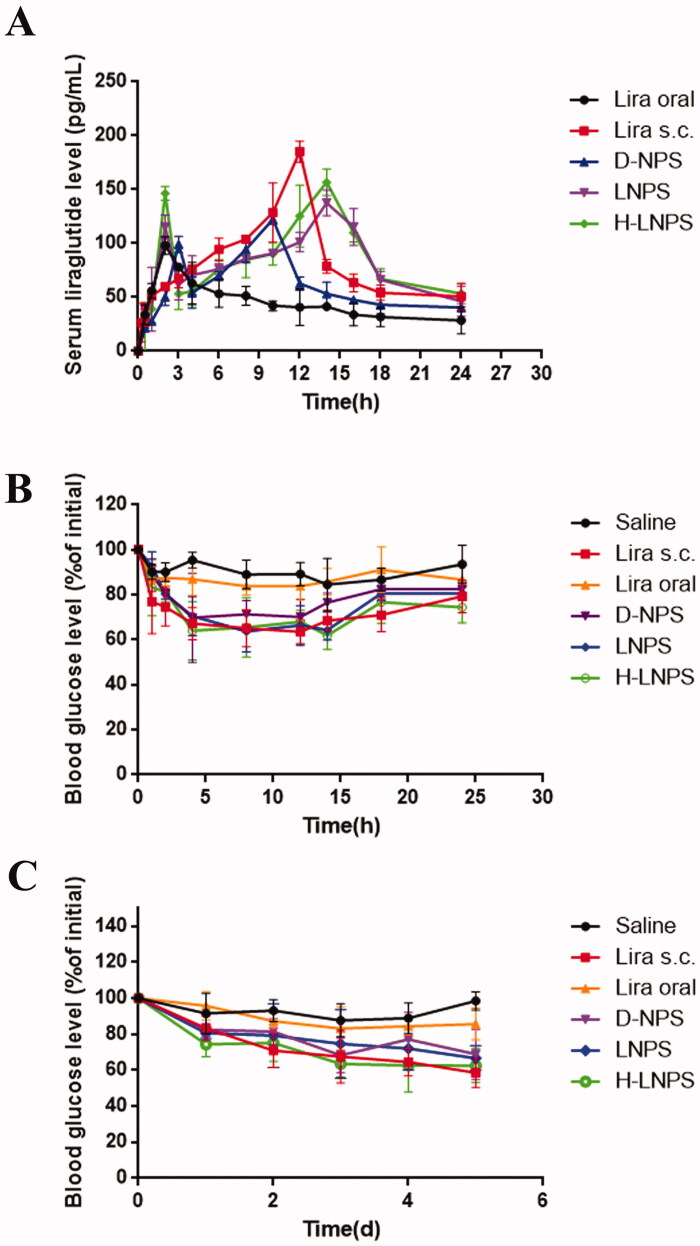
(A) Blood concentration of liraglutide at different times. Blood glucose levels in diabetic mice following single administration (B) and multiple administrations (C). Lira oral refers to the oral liraglutide solution group and Lira s.c. refers to the subcutaneous injection group.

The results of the pharmacokinetic study showed that the bioavailability of D-NPS, LNPS, and H-LNPS was higher than that of the oral liraglutide group, indicating that liraglutide could improve bioavailability after carrier coating, and the self-ablating nanoparticles group were higher than the self-assembled nanoparticles (Table 3). The results of the pharmacodynamic study showed (Figure 7(B,C)) that saline and liraglutide solution could not lower blood glucose, while D-NPS, LNPS, and H-LNPS could achieve some hypoglycemic effect, and the hypoglycemic effect of the H-LNPS group was the best, and the difference with subcutaneous injection group was also small. The bioavailability and hypoglycemic effect of H-LNPS than in LNPS, indicating that the promotion of nanoparticle lysosome escape could improve the bioavailability and hypoglycemic effect of liraglutide.

## Conclusion

Self-ablating nanoparticles with core–shell structures (LNPS) were prepared following a combination of the emulsifying-volatilization and thin-film dispersion methods. H-LNPS was prepared by loading HA_2_, and D-NPS was prepared following the self-assembly method. The results revealed that self-ablating nanoparticles exhibited higher encapsulation rates and better stability than self-assembling nanoparticles. Multiple internalization pathways can be followed for the internalization of the self-ablating nanoparticles, which exhibited good extents of cellular uptake. HA_2_ loading facilitates the escape of LNPS from lysosomes. The small self-assembling nanoparticles exhibit a good extent of intestinal uptake in the presence of mucus. The bioavailability and hypoglycemic effect of self-ablating nanoparticles were also better than that of self-assembled nanoparticles. The results reported herein confirm that zwitterionic materials can be used to achieve a high extent of cellular uptake. It was also observed that they exhibit good intestinal absorption potential.
